# Prevalence and Characterization of Food-Borne *Vibrio parahaemolyticus* From African Salad in Southern Nigeria

**DOI:** 10.3389/fmicb.2021.632266

**Published:** 2021-06-08

**Authors:** Etinosa O. Igbinosa, Abeni Beshiru, Isoken H. Igbinosa, Abraham G. Ogofure, Kate E. Uwhuba

**Affiliations:** ^1^Applied Microbial Processes and Environmental Health Research Group, Faculty of Life Sciences, University of Benin, Benin City, Nigeria; ^2^Department of Microbiology, College of Natural and Applied Sciences, Western Delta University, Oghara, Nigeria

**Keywords:** African salad, vibrios, vegetables, food safety, retail outlets, epidemiology

## Abstract

The demand for minimally processed vegetables (African salad) has increased partly due to its inclusion in ready-to-eat foods. Nevertheless, the associated risk of the presence of emergent foodborne pathogens, such as *Vibrio parahaemolyticus* might be underestimated. The present study was designed to isolate and characterize foodborne *V. parahaemolyticus* from minimally processed vegetables using culture-based methods and molecular approach. A total of 300 samples were examined from retail outlets between November 2018 and August 2019 from Southern Nigeria. The prevalence of vibrios from the overall samples based on the colonial proliferation of yellow, blue-green and/or green colonies on thiosulfate citrate bile salts sucrose agar was 74/300 (24.6%). An average of two green or blue-green colonies from respective plates was screened for *V. parahaemolyticus* using analytical profile index (API) 20 NE. Polymerase chain reaction further confirmed the identity of positive *V. parahaemolyticus*. The counts of *V. parahaemolyticus* ranged from 1.5 to 1,000 MPN/g. A total of 63 recovered *V. parahaemolyticus* were characterized further. The resistance profile of the isolates include ampicillin 57/63 (90.5%), cefotaxime 41/63 (65.1%), ceftazidime 30/63 (47.6%), amikacin 32/63 (50.8%), kanamycin 15/63 (23.8%), and oxytetracycline 16/63 (25.4%). The multiple antibiotic index ranged from 0–0.81. The formation of biofilm by the isolates revealed the following: strong formation 15/63 (23.8%), moderate formation 31/63 (49.2%), weak formation 12/63 (19.1%), and no formation 5/63 (7.9%). A total of 63/63 (100%), 9/63 (14.3%), and 20/63 (31.8%) of the isolates harbored the *tox R* gene, TDH-related hemolysin (*trh*) and thermostable direct hemolysin (*tdh*) determinants respectively. The isolates with O2 serogroup were most prevalent *via* PCR. Isolates that were resistant to tetracycline, kanamycin, and chloramphenicol possessed resistant genes. The presence of multidrug-resistant vibrios in the minimally processed vegetables constitutes a public health risk and thus necessitates continued surveillance.

## Introduction

*Vibrio parahaemolyticus* is a Gram-negative halophilic bacterium distributed in estuarine and marine environments (Wu et al., 2014). Significant proportions of *V. parahaemolyticus* strains recovered from environmental sources are commensals of marine microbiota, with a few fractions of disease-causing *V. parahaemolyticus* strains. These bacterial infections are commonly connected with food-borne gastroenteritis (Lopatek et al., 2018). The virulent strains of *V. parahaemolyticus* are differentiated from the non-virulent strains by their capability to possess and express toxigenic genes such as, tdh-related (*trh*) hemolysin and/or thermostable direct hemolysin (*tdh*) genes (Letchumanan et al., 2014). The *trh* and *tdh* determinants are referred to as important virulence elements of *V. parahaemolyticus* (Xu et al., 2014). *V. parahaemolyticus* is often isolated from food with most of the isolates not harboring or expressing pathogenic potentials and hence, less detrimental to humans (Velazquez-Roman et al., 2012). It has been reported by Gutierrez-West et al. (2013) that *V. parahaemolyticus* isolated from environmental samples don’t usually cause diseases in marine animals and humans.

Different approaches for the detection and/or isolation of *V. parahaemolyticus* have been reported. However, the standard procedure for the isolation of *V. parahaemolyticus* employs the use of enrichment media such as alkaline peptone water and selective media such as thiosulfate-citrate-bile salts-sucrose agar, along with some biochemical tests (Vincent et al., 2015). These methods are essential for the quantification of *V. parahaemolyticus* density in a sample. This aids potential risk estimation for the occurrence of pathogenic strains (Malcolm et al., 2015). Rapid and accurate *V. parahaemolyticus* identification combines both molecular and conventional approaches. *V. parahaemolyticus* pathogens from food samples are detected using polymerase chain reaction methods which amplifies the toxin operon gene (*tox*-R) sequences (Law et al., 2015).

Genes responsible for antibiotic resistance (AR) in vibrios are localized in plasmids or chromosomes. Extra-chromosomal genetic materials are crucial mediators responsible for the spread of antibiotic-resistant genes (ARGs) and can be interchanged with other bacteria *via* horizontal and vertical gene transfer (Manjusha and Sarita, 2011). Biofilms are architecturally complex assemblies of microorganisms on or in biotic or abiotic surfaces and interfaces, characterized by interactions between the populations. Biofilms contain exopolymeric substances and survive as self-organized, three-dimensional structures that exhibit altered phenotypic and genotypic characters (Mizan et al., 2015). *V. parahaemolyticus* is known to form biofilms on food and food contact surfaces. Like other biofilm-producing microorganisms, *V. parahaemolyticus* is capable of producing distinct types of adherence factors that enable the bacterium to adhere to the surface and initiate biofilm formation (Donlan, 2002). Bacterial biofilm potentials can support resistance to some physical and/or chemical agents thereby improving their resistance profile (Beshiru and Igbinosa, 2018). Formation and development of biofilm is complex and heterogeneous which makes phenotypic readouts acceptable as standards for evaluating the impacts of biofilm formation on antibiotics.

African salad contains lots of raw and fresh vegetables attached to some other ingredients that are eaten without further processing, which makes it a salad of African origin. African salad is generally sourced for due to its constituents. It is known to be rich in carbohydrate, protein, minerals, and vitamins. African salad is often consumed as a meal or as an alternate dish to the numerous Nigerian food recipes (Maky, 2013). Although Nigeria has no official foodborne disease surveillance system, foodborne infection is endemic in Nigeria and its direct cost is estimated at $3 billion, accounting for 17–25% of the estimated cost of all illnesses (Oludare et al., 2016). The local government health system profile in Nigeria revealed food-borne illnesses as the leading cause of death, accounting for 25% mortality. An outbreak of food poisoning in Ibadan, Nigeria, claimed about 20 lives (Osagbemi et al., 2010). Cases of food poisoning among families in Kano and Ilorin Nigeria due to consumption of yam flour have been documented (Adedoyin et al., 2008; Adeleke, 2009). An acute onset of gastrointestinal symptoms among people who had attended and eaten at a burial ceremony resulted in 60 case patients and three deaths (Fatiregun et al., 2010). The World Health Organization (WHO) estimates that >200,000 people die of food poisoning annually in Nigeria from foodborne pathogens. The deaths were caused by contaminated foods through improper processing, preservation and service World Health Organization [WHO], 2009.

In Nigeria, a large proportion of ready-to-eat foods are sold by the informal sector, especially as street foods. The hygienic aspects of vending operations and the safety of these foods are problematic for food safety regulators. The global food crisis has worsened an already precarious food situation because when food is in short supply and/or expensive, people are more concerned about satisfying hunger than the safety of the food. It has been revealed that in Nigeria, 27.7% of food handlers do not wash their hands before preparing food and 28.1% use only water to wash hands after using the toilet. The knowledge of food handlers about the food borne infections (FBIs) and their safety practices is an important issue in the outbreaks of FBI. It was also revealed that 90% of food handlers have heard about foodborne infection out of which only 15.6% of them know how it is contracted (Smith et al., 2010). Furthermore, large quantity of food produced and distributed gets to the consumers in an unwholesome condition due to poor handling methods, inefficient processing equipment and storage practices, high ambient tropical temperature and humidity conditions (Akintaro, 2012).

*Vibrio parahaemolyticus* have been recovered from seafoods in different parts of Nigeria (Adebayo-Tayo et al., 2011; Oramadike and Ogunbanwo, 2015; Beshiru et al., 2020). Other bacteria (such as *P. aeruginosa*, *P. putida*, *P. mendocina*, *E. coli*, *Staphylococcus* spp., *Pseudomonas* spp., *C. septicum*, *Micrococcus* spp., and *B. alvei*, *Enterococcus* spp., *K. pneumoniae*, *Salmonella* spp., *Shigella* spp., *Citrobacter* spp., *B. thuringiensis*, *B. subtilis*, and *B. licheniformis*) have been recovered from other ready-to-eat foods in Nigeria which includes Buns, Meat pie, Egg roll, Wara, Kununzaki, Suya, Rice, Beans, Bread, Akara, Potato, and Yam (Bukar et al., 2009; Adio et al., 2014; Agwa et al., 2014; Madueke et al., 2014; Aruwa and Ogunlade, 2016; Fadahunsi and Makinde, 2018; Obande et al., 2018; Igbinosa et al., 2020; Beshiru et al., 2021). Oguwike et al. (2014) detected *Salmonella* spp., *Shigella* spp., and *S. aures* from African salads in Enugu Nigeria. The predominant bacterial isolates from African salad by Oranusi et al. (2013b) in Owerri Imo State Nigeria belong to *Bacillus* spp., *Staphylococcus* spp., *Escherichia coli*, Enterococci and *Serracia* spp. The species of bacteria isolated by Daniel et al. (2016) from African salads in Benin City Edo State were identified as *Listeria* sp., *E. coli*, *Klebsiella* sp., *Proteus* sp., *Salmonella* sp., *Staphylococcus aureus*, and *Shigella* sp. Furthermore, *Proteus vulgaris*, *S. aureus*, *Citrobacter freundii*, *Proteus mirabilis*, and *Corynebacterium* spp. were recovered from pre-packed mixed vegetable salad within Lagos metropolis Nigeria (Uzeh et al., 2009). *Acinetobacter baumannii*, *Bacillus* sp., *Enterobacter* sp., *E. coli*, *Klebsiella* sp., *Kurthia gibsonii*, and *Salmonella* sp. were recovered from commonly vended African Salads in Owerri Imo State Nigeria (Dike et al., 2017). Due to lack of information concerning the detection and characterization of *V. parahaemolyticus* from African salads sold in retail outlets despite their increased consumption, we aimed to investigate *V. parahaemolyticus* from African salads.

Gastroenteritis outbreaks which result from *V. parahaemolyticus* have been reported globally, such as in Japan (Arakawa et al., 1999), Europe (Lopatek et al., 2018), South America (Raszl et al., 2016), and United States (Shaw et al., 2014). Due to the poor repository and documentation from food outbreaks in Nigeria, it is difficult to show statistics of *V. parahaemolyticus* outbreaks. However, to date, there is limited literature on the contamination levels, prevalence, and pathogenic profiles of *V. parahaemolyticus* from African salads. Hence, we presented information indicating the importance of coordinated detection and characterization of *V. parahaemolyticus* strains from African salads.

## Materials and Methods

### Sample Collection

The study focused on African salads from eight States in southern Nigeria (Edo State, Delta State, Imo State, Anambra State, Enugu State, Abia State, Ondo State, and Lagos State). Sample size determination formula was used in this study to determine the sample size as follows:

$$\text{Sample}\,\left(\text{N}\right)=\frac{{\left({\text{Z}}_{1-\alpha /2}\right)}^{2}\,P\,\left(1-\text{P}\right)}{d^{2}}$$

Z_1-α/2_ = Standard normal variant at 5% type I error (*P* < 0.05); P = Expected prevalence based on previous study [20.65% from Tunung et al. (2010); 8.4% from Kriem et al. (2015); 15% from Obaidat et al. (2017) were used]; d = Absolute error or precision (which is 5%). Hence, the expected sample size was a minimum of 118 samples. A total of 300 African salad samples were thereafter randomly purchased from retails outlets from respective eight (08) states covering the southern region of Nigeria between November 2018 and August 2019. All the samples were sealed and conveyed in an icebox to the Applied Microbial Processes and Environmental Health Research (AMPEHREG) laboratory for analysis within 12 h of sample collection.

### Enumeration and Isolation of *Vibrio parahaemolyticus* From the African Salad Samples

Twenty-five (25) grams of African salad samples were weighed and immersed into a sterile homogenizer bag which contains 225 mL of alkaline peptone water (Lab M, Lancashire, United Kingdom), pH 8.5, 2% NaCl, giving a first-order dilution (10^–1^). The samples were homogenized for 1 min at 800 rpm using a shaker. A 3 × 10 mL portion of the 1:10 dilution which represents the 1 g portion was inoculated into three tubes containing 10 mL of 2 × alkaline peptone water. Similarly, 3 × 1 mL portions of the 1:10, 1:100, and 1:1,000 dilutions were inoculated into 10 mL of single-strength alkaline peptone water and incubated overnight at 35 ± 2°C. Streak plate technique using a 3-mm loopful from the top 1 cm of the alkaline peptone water tubes containing the highest dilutions of the sample showing growth was inoculated onto thiosulphate-citrate-bile salt-sucrose (TCBS) agar (Lab M, Lancashire, United Kingdom) and incubated at 35 ± 2°C overnight. *V. parahaemolyticus* appear as 2–3 mm in diameter, opaque, round, bluish-green or green colonies, on TCBS agar. A minimum of two and maximum of five colonies were selected for identification. The biochemical and morphological test employed includes: urease test using Christensen’s urea agar (Lab M, Lancashire, United Kingdom) supplemented with 2% NaCl, 3% KOH, arginine glucose slant test, motility test, oxidase test, 3.5% NaCl triple-sugar-iron, Gram staining, ortho-Nitrophenyl-β-galactoside (ONPG) test, sugar fermentation test (lactose and cellobiose reactions), T_1_N_0_ and T_1_N_3_ broths (QingDao Hopebio-Technology, China) test and salt tolerance test. Cell suspensions of tentative *V. parahaemolyticus* in 2% NaCl were further screened using analytical profile index (API) 20NE. The tests were performed according to the manufacturer’s instruction for use. Data interpretation was carried out using the API database (V4.1) with the apiweb^TM^ identification software. Confirmed isolates were purified on tryptone soy agar (Lab M, Lancashire, United Kingdom) supplemented with 2% NaCl and stored in nutrient agar (Lab M, Lancashire, United Kingdom) slants containing 2% NaCl at 4°C. *V. parahaemolyticus* colonies were expressed in MPN/g.

### Identification of *Vibrio parahaemolyticus* and Detection of Virulence Genes

Pure isolates where DNA was extracted were resuscitated on tryptone soy broth (Lab M, Lancashire, United Kingdom) and incubated for 18 h at 37°C. DNA isolation was carried out using PeqGold Bacterial DNA kit (Peqlab Biotechnologie GmbH, Germany) following the manufacturers’ instruction. PCR amplification for detection of *tox*-R, *trh* and *tdh* elements was carried out *via* PCR using specific primers and annealing conditions stated in Supplementary Table 1 below. This was conducted using a final volume of 20 μL, containing 10 μL of 2 × Taq polymerase, MasterMix, 2 μL of DNA template, 1 μL each primer concentration of 05 μM, and 6 μL of sterile distilled water. The PCR amplifications were performed using a Peltier-Based Thermal Cycler (BioSeparation System, China) with the following cycling conditions: initial denaturation for 3 min at 94°C, 30 cycles for 1 min at 94°C, 1 min at 58°C and 1 min at 72°C, and a final elongation for 5 min at 72°C. All PCR products were visualized by using 1.0% agarose gel (CLS-AG100, United Kingdom) in 0.5 × TAE buffer and allowed to run for 1 h at 100 V. The gels were visualized using UV transilluminator (EBOX VX5; France). *V. parahaemolyticus* DSM 11058 was used as a positive control and double-distilled water (ddH_2_O) as a negative control in every PCR reactions.

### Biofilm Formation, Hemolytic Response and Screening for O Serogroups

Biofilm formation was assayed *via* the microtiter plate method as described previously by Beshiru et al. (2018). Wagatsuma agar (HiMedia, India) was used to elucidate hemolytic response by adopting the Kanagawa phenomenon. The serogroups of *V. parahaemolyticus* isolates were identified *via* PCR technique using primer sets and amplification conditions as previously described (Chen et al., 2012). *V. parahaemolyticus* DSM 11058 was used as a positive control and ddH_2_O as a negative control in all PCR reactions.

### Antimicrobial Susceptibility Profile of the Isolates

Antimicrobial susceptibility profile of the *V. parahaemolyticus* isolates was performed using Kirby–Bauer disk diffusion method and readings interpreted by adopting the breakpoints of Clinical and Laboratory Standard Institute. Briefly, purified isolates were inoculated on 5 mL Mueller Hinton broth (Lab M, United Kingdom) and incubated overnight. The OD of the broth was determined to be equivalent to × 10^6^ CFU/mL. With sterile swab sticks, respective standards were aseptically swabbed on Mueller Hinton agar (Lab M, United Kingdom). Antibiotics employed in this study include the recommended antibiotics by the Centre for Disease Control and Prevention for the treatment of vibrios infections (Shaw et al., 2014). A total of 19 antibiotic disks (Mast Diagnostics, United Kingdom) which includes: ceftazidime (30 μg), ampicillin (10 μg), imipenem (10 μg), cefotaxime (30 μg), amikacin (30 μg), ampicillin/sulbactam (10/10 μg), cefazolin (30 μg), cephalothin (30 μg), gentamicin (10 μg), azithromycin (15 μg), chloramphenicol (30 μg), ciprofloxacin (5 μg), levofloxacin (5 μg), kanamycin (30 μg), streptomycin (10 μg), nalidixic acid (30 μg), tetracycline (30 μg), oxytetracycline (30 μg), and trimethoprim-sulfamethoxazole (1.25/23.75 μg). The respective disks were also impregnated aseptically on the agar plates equidistant apart and incubated at 37°C for 18–24 h. Characterization of the resistance (R), intermediate (I) or susceptibility (S) profile of the isolates was determined by measuring inhibitory zones and then compared with the interpretative chart to determine the sensitivity, intermediate and resistant nature of the isolates to the antibiotics used using the interpretative chart by Clinical and Laboratory Standard Institute [CLSI] (2018). Multiple antibiotic resistance index (MARI) and multidrug resistance (MDR) were determined as described previously (Igbinosa and Beshiru, 2019).

### Screening for Antibiotic Resistance Genes

*Vibrio parahaemolyticus* isolates that revealed resistance phenotypes were screened for corresponding resistance elements. The elements associated with resistance to sulfamethoxazole (*sul3, sul2*, and *sul1*), kanamycin (*aphA-3*), chloramphenicols (*catA1, catA2, catA3*, and *catB3*), trimethoprim (*dfr*), tetracyclines (*tetM, tetG, tetC, tetB*, and *tetA*), and β-lactams (*blaOXA, blaSHV*, and *blaTEM*) were detected by PCR (Kim et al., 2013). ARGs primers are listed below (Supplementary Table 1). The amplification procedure was performed using a Peltier-Based Thermal Cycler (BioSeparation System, Shanxi, China) with the following cycling conditions: initial denaturation at 94°C for 3 min, 35 cycles at 94°C for 30 s, 1 min at 52°C and 90 s at 72°C, and final elongation at 72°C for 6 min. All PCR products were visualized by using 1.5% agarose gel (CLS-AG100, United Kingdom) in 0.5 × TAE buffer and run for 1 h at 100 V. The gels were visualized using UV transilluminator (EBOX VX5; France).

### Statistical Analysis

Data were analyzed using the SPSS statistical software (Version 21.0). Descriptive statistics were used to estimate the mean and standard deviation. Correlation analysis was used to elucidate the impact of one virulence factor/determinant on another. The *p*-values < 0.05 were considered significant statistically.

## Results

### Prevalence and Population Cell Density of *Vibrio parahaemolyticus* From African Salads

**FIGURE 1:**
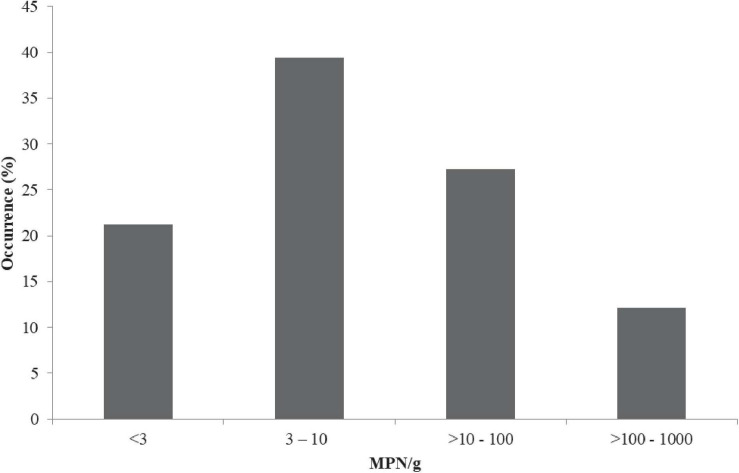
Levels of *V. parahaemolyticus* in African salads. MPN/g, most probable number per gram; <, less than; >, greater than.

The prevalence of vibrios from the overall samples based on the colonial occurrence of yellow, green and/or blue-green colonies on TCBS agar was 74/300 (24.6%). The prevalence of *V. parahaemolyticus via* API 20NE and PCR was 33/300 (11%). The cell density of *V. parahaemolyticus* ranged from 1.5 to 1,000 MPN/g (Figure 1). From the positive samples (samples which harbored *V. parahaemolyticus*), 7/33 (21.21%) contained bacterial load < 3 MPN/g, 13/33 (39.39%) contained a load 3–10 MPN/g, 9/33 (27.27%) contained > 10–100 MPN/g, and 4/33 (12.12%) samples contained > 100–1,000 MPN/g (Figure 1). Statistically, a significant difference exists in the *V. parahaemolyticus* density based on samples from different locations (states) (*p* < 0.05). A total of 63 isolates were recovered. The distribution of the 63 *V. parahaemolyticus* isolates is as follows: Edo State 7/63 (11.11%), Delta State 10/63 (15.87%), Imo State 9/63 (14.29%), Anambra State 8/63 (12.69%), Enugu State 9/63 (14.29%), Abia State 7/63 (11.11%), Lagos State 8/63 (12.69%), and Ondo State 5/63 (7.94%).

### Prevalence of Phenotypic and Genotypic Virulence Profile of *Vibrio parahaemolyticus* From African Salads

**FIGURE 2:**
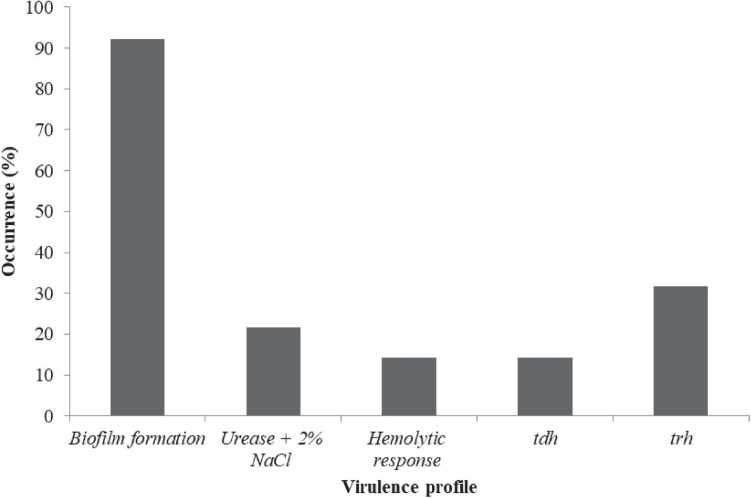
Phenotypic and genotypic virulence profile of *V. parahaemolyticus* from African salads. Thermostable direct hemolysin (*tdh*) and TDH-related hemolysin (*trh*).

**FIGURE 3:**
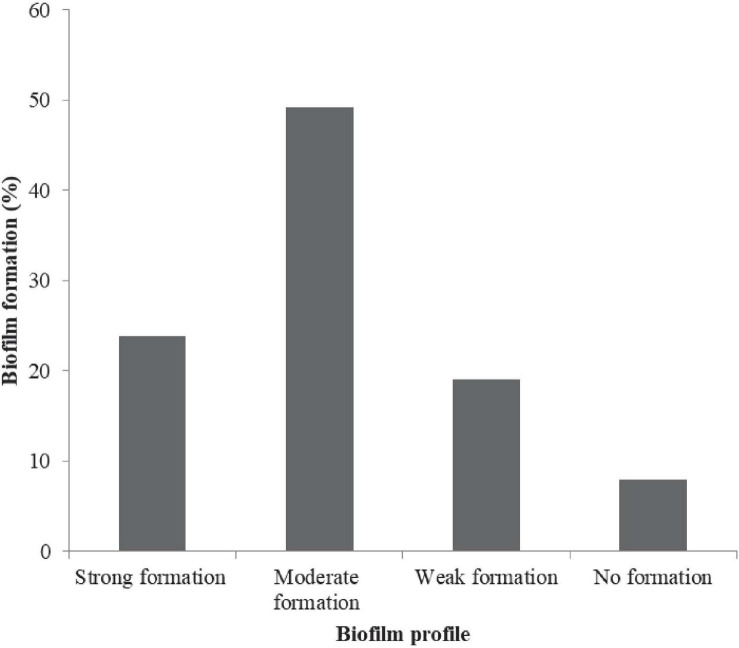
Biofilm formation profile of *V. parahaemolyticus* from African salads.

**FIGURE 4:**
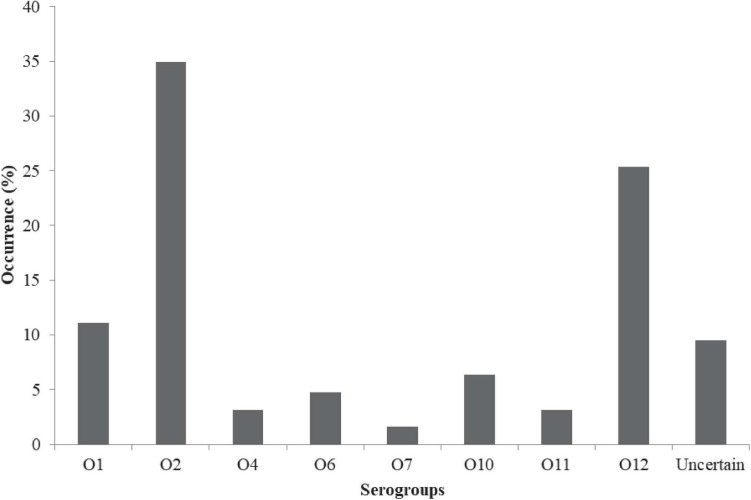
Distribution of serogroups of *V. parahaemolyticus* from African salads.

The prevalence in Figure 2 include: Urease + 2% NaCl 20/63 (21.75%) and hemolytic response 9/63 (14.29%). A total of 63/63 (100%), 9/63 (14.3%), and 20/63 (31.8%) of the isolates harbored the *tox R*, *tdh*, and *trh* genes, respectively (Figure 2). Urease + 2% NaCl significantly correlated hemolytic response (*r* = 0.599, *p* < 0.01), *tdh* genes (*r* = 1.000, *p* < 0.01) and *trh* genes (*r* = 0.599, *p* < 0.01) as shown in Supplementary Table 3. In addition, *tdh* genes significantly correlated *trh* genes (*r* = 0.599, *p* < 0.01). The biofilm formation profile in Figure 3 revealed the following: strong formation 15/63 (23.8%), moderate formation 31/63 (49.2%), weak formation 12/63 (19.1%), and no formation 5/63 (7.9%). Overall, 58/63 (92.1%) of the isolates formed biofilm. Biofilm formation significantly correlated Urease + 2% NaCl (*r* = 0.686, *p* < 0.01), hemolytic response (*r* = 0.424, *p* < 0.01), *tdh* genes (*r* = 0.424, *p* < 0.01), and *trh* genes (*r* = 0.686, *p* < 0.01). The prevalence of the serogroups in Figure 4 is as follows: O1 7/63 (11.11%), O2 22/63 (34.92%), O4 2/63 (3.17%), O6 3/63 (4.76%), O7 1/63 (1.59%), O10 4/63 (6.35%), O11 2/63 (3.18%), O12 16/63 (25.39%), and uncertain 6/63 (9.52%). Location and serogroups had no correlation on the phenotypic and/or genotypic profile of the isolates (Supplementary Table 3).

### Antimicrobial Susceptibility Profile of *Vibrio parahaemolyticus* Isolates From African Salads

**FIGURE 5:**
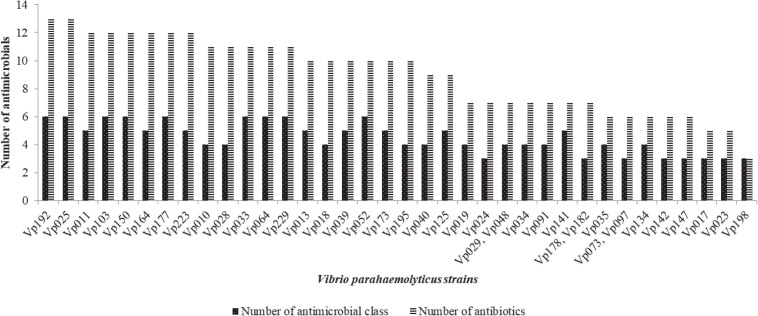
The MDR profile of *V. parahaemolyticus* from African salads.

**FIGURE 6:**
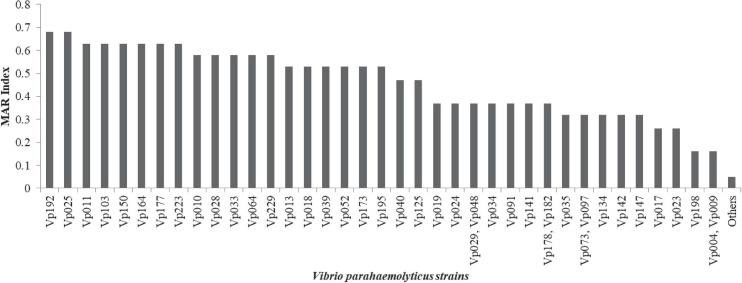
MAR index of *V. parahaemolyticus* recovered from African salads. Others = Vp014, Vp026, Vp038, Vp043, Vp053, Vp056, Vp072, Vp111, Vp149, Vp165, Vp172, Vp175, Vp180, Vp185, Vp194, Vp196, Vp224, and Vp152.

Antimicrobial susceptibility profile of *V. parahaemolyticus* from African salads in Table 1 is as follows: ampicillin 57/63 (90.5%), cefotaxime 41/63 (65.1%), cephalothin 37/63 (58.7%), amikacin 32/63 (50.8%), ceftazidime 30/63 (47.6%), cefazolin 29/63 (46.0%), streptomycin 19/63 (30.2%), oxytetracycline 16/63 (25.4%), kanamycin 15/63 (23.8%), ciprofloxacin 10/63 (15.9%), tetracycline 12/63 (19.1%), and trimethoprim-sulfamethoxazole 10/63 (15.9%). High level of sensitivity observed includes nalidixic acid 62/63 (98.4%), imipenem 54/63 (85.7%), ciprofloxacin 49/63 (77.8%), azithromycin 49/63 (77.8%), levofloxacin 48/63 (76.2%), gentamicin 46/63 (73.0%), tetracycline 43/63 (68.3%), and trimethoprim-sulfamethoxazole 35/63 (55.6%). The MDR profile in Figure 5 revealed that 39/63 (61.9%) of the isolates had MDR potential (resistant to ≥3 antibiotics in ≥3 antimicrobial class). The MAR index ranged from 0.05–0.68 (Figure 6) with 35/63 (53.8%) of the isolates having MAR index > 0.2. A total of 4/63 (6.3%) isolates were not resistant to any of the antibiotics used with MAR index of 0.00 while 18/63 (28.6%) of the *V. parahaemolyticus* were resistant to one antibiotic with MAR index of 0.05. A total of 3/63 (4.8%) of the *V. parahaemolyticus* were resistant to three antibiotics with MAR index of 0.16. The MAR index significantly correlates the formation of biofilm (*r* = 0.474, *p* < 0.01).

**TABLE 1:** Antibiotic susceptibility profile of the *V. parahaemolyticus* isolates.

**Antimicrobial class**	**Antibiotics**	***V. parahaemolyticus* (*n* = 63)**		
		**Resistance**	**Intermediate**	**Sensitive**
Penicillins	Ampicillin/sulbactam (10/10 μg)	18 (28.6)	12 (19.0)	33 (52.4)
	Ampicillin (10 μg)	57 (90.5)	6 (9.5)	0 (0)
Aminoglycosides	Amikacin (30 μg)	32 (50.8)	21 (33.3)	10 (15.9)
	Gentamicin (10 μg)	6 (9.5)	11 (17.5)	46 (73.0)
	Kanamycin (30 μg)	15 (23.8)	25 (39.6)	23 (36.5)
	Streptomycin (10 μg)	19 (30.2)	32 (50.8)	12 (19.1)
Carbapenems	Imipenem (10 μg)	2 (3.2)	7 (11.1)	54 (85.7)
Cephalosporins	Cefotaxime (30 μg)	41 (65.1)	9 (14.3)	13 (20.6)
	Ceftazidime (30 μg)	30 (47.6)	12 (19.1)	21 (33.3)
	Cephalothin (30 μg)	37 (58.7)	11 (17.5)	15 (23.8)
	Cefazolin (30 μg)	29 (46.0)	NA	34 (54.0)
Quinolones	Nalidixic acid (30 μg)	1 (1.6)	0 (0)	62 (98.4)
	Levofloxacin (5 μg)	9 (14.3)	6 (9.5)	48 (76.2)
	Ciprofloxacin (5 μg)	10 (15.9)	4 (6.3)	49 (77.8)
Phenicols	Chloramphenicol (30 μg)	14 (22.2)	23 (36.5)	26 (41.3)
Folate pathway inhibitor	Trimethoprim-sulfamethoxazole (1.25/23.75 μg)	10 (15.9)	18 (28.6)	35 (55.6)
Tetracyclines	Tetracycline (30 μg)	12 (19.1)	8 (12.7)	43 (68.3)
	Oxytetracycline (30 μg)	16 (25.4)	25 (39.6)	22 (34.9)
Macrolides	Azithromycin (15 μg)	9 (14.3)	5 (7.9)	49 (77.8)

### Antibiotic Resistance Genes in the *Vibrio parahaemolyticus*

**FIGURE 7:**
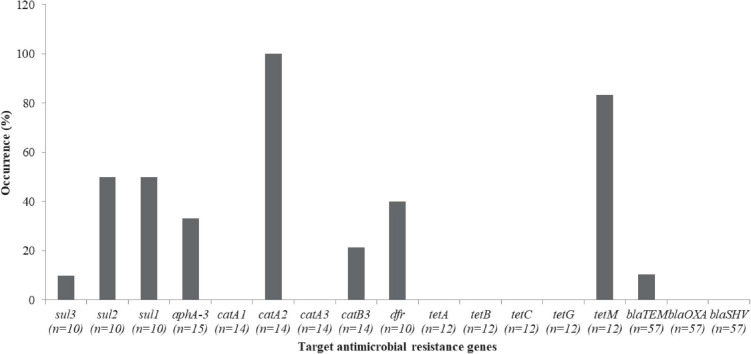
Antibiotic resistance genes in the *V. parahaemolyticus*.

*Vibrio parahaemolyticus* isolates that were resistant to kanamycin (*n* = 15), chloramphenicol (*n* = 14), tetracycline (*n* = 12), trimethoprim-sulfamethoxazole (*n* = 10) and β-lactam (*n* = 57), were screened for the occurrence of their respective resistance genes [kanamycin (*aphA*-3), chloramphenicol (*catA2*, *catA1*, *catB3*, and *catA3*), tetracycline (*tetA*, *tetB*, *tetC*, *tetG*, and *tetM*), trimethoprim (*dfr*), sulfamethoxazole (*sul3*, *sul2*, and *sul1*), and β-lactam (*blaTEM*, *blaOXA* and *blaSHV*)]. Resistance genes such as *sul3* 1/10 (10%), *sul2* 5/10 (50%), and *sul1* 5/10 (50%) were detected from the sulfamethoxazole resistance (Figure 7). In addition, 5/15 (33.3%) of *aphA-3* and 4/10 (40%) of *dfr* of *V. parahaemolyticus* isolates were detected. Others include *catA2* 14/14 (100%), *catB3* 3/14 (21.4%), *tetM* 10/12 (83.3%), and *blaTEM* 6/57 (10.5%). *catA1, catA3, tetA, tetB, tetC, tetG, blaOXA*, and *blaSHV* were not detected (Figure 7).

## Discussion

The occurrence of disease-causing *V. parahaemolyticus* strains in salads is of immense concern to public health as this is a frequent causal organism of food-borne gastroenteritis in humans. The highest microbial density of 3–10 MPN/g (13/33; 39.39%) observed in this study is familiar to the study of Yang et al. (2017) and Xu et al. (2016). However, it was higher compared to the finding of Tunung et al. (2010). The level of *V. parahaemolyticus* prevalence in this study appears to be lower when compared to the findings of Lee et al. (2018) in which 69% (165/240) of the analyzed samples were known to be positive to *V. parahaemolyticus* (based on *toxR* PCR assay). Similarly, the study of Tan et al. (2017) and Narayanan et al. (2020) showed the prevalence of *V. parahaemolyticus* in samples analyzed as 79.5 and 90% respectively. Contrarily, the study of Yang et al. (2017) demonstrated a lower level *V. parahaemolyticus* prevalence as 19.44% in tested samples. African salad is a ready-to-eat food therefore the presence of pathogenic *V. parahaemolyticus* (>100–1,000 MPN/g) in this study is of paramount public health concern.

The detection of *V. parahaemolyticus* in African salad could be as a result of the materials used in the preparation process, notably the vegetable, water, and seafood. The previous report by Eni et al. (2010) has implicated vegetables as a veritable source of microbial pathogens. In further agreement with this study, Eni et al. (2010); Tunung et al. (2010); and Oranusi et al. (2013b) reported the presence of microbial contamination in African salad. Oranusi et al. (2013a) further emphasize the hazard resulting from the consumption of undercooked and moderately cooked food as they are likely to retain more pathogens even though they could contain more nutrients. Increase in FBI has been linked with the lack of knowledge of FBIs and poor food safety practice (FSP) of food handlers. This is a major determinant of the kind of patronage received by fast food and local restaurants. Inadequate food safety laws, weak regulatory systems, lack of financial resources to invest in safer equipment, inadequate knowledge of food borne diseases and their causes, improper handling of food and unhygienic environments among others have been identified as some of the causes of FBI (Haileselassie et al., 2013).

*Vibrio parahaemolyticus* strains from food samples have been observed to not usually harbor pathogenic genes *trh* and/or *tdh* which accounts for virulence traits of such strains in human (Gutierrez-West et al., 2013). Similarly, it is of note that *V. parahaemolyticus* recovered from the food and environment contains much less pathogenic strains than clinical isolates (Rahimi et al., 2010). Previous studies equally reported that only 1–2% of the environmental *V. parahaemolyticus* strains usually carry the *tdh* and/or *trh* genes (Velazquez-Roman et al., 2012; Haley et al., 2014). Xu et al. (2014) considered these two genes as the principal virulence elements of *V. parahaemolyticus.* However, some clinical *V. parahaemolyticus* isolates have also been stated not to possess pathogenic characteristics (Xu et al., 2016). Hence where these two hemolysins (*tdh* and *trh*) are absent, *V. parahaemolyticus* can remain pathogenic, portraying the existence of other virulence determinants/factors. Hence, the need to investigate other *V. parahaemolyticus* virulence factors such as urease activity, β-hemolytic activity and biofilm formation. With the urease enzyme, urea is hydrolyzed into carbon dioxide and ammonia, raising the pH in the immediate environment inside the host or habitat. This microhabitat activity contributes to survival in the digestive tract, inflammation, blood vessel permeability and tissue invasion (Berutti et al., 2014).

Early studies showed that urease induces the accumulation of intestinal fluid in the rabbit ileal loops test and causes gastrointestinal inflammatory lesions, confirming that urease is an important virulence factor in *trh* positive *V. parahaemolyticus* strains (Cal and Ni, 1996; Osawa et al., 1996; Wang et al., 2015). Findings from our study agree with a previous report by Lopatek et al. (2018) that *trh*-positive *V. parahaemolyticus* had urease production potential. This is also similar to the report of Quadri et al. (2005) that *trh*-positive *V. parahaemolyticus* strains almost always correlate with urease production. In our study, production of urease correlated with the occurrence of *trh* and *tdh* genes. Sujeewa et al. (2009) reported that not all urease positive strains of *V. parahaemolyticus* were positive for either *tdh* or *trh* determinants. However, Sujeewa et al. (2009) revealed that *tdh* positive isolates significantly correlated with isolates exhibiting hemolysis, thereby implicating hemolytic activity as a strong indicator for *tdh* genes. This tallies with the results of this study whereby all isolates that exhibited hemolysis completely correlated with the presence of *tdh* genes. Though urease production and hemolytic response could be considered as a notable marker of *trh* and *tdh* in *V. parahaemolyticus*, they are not accurate as a pathogenic biomarker. Findings on the incidence of *tox-R* gene in this study are in line with the report of Yang et al. (2017) in which all the tested isolates were also positive for *tox*-R gene. African salad is at high risk of *V. parahaemolyticus* infections among the foods available in Nigeria sequel to the high pathogenic strains recovered.

The previous study by Letchumanan et al. (2014) attributed *Vibrio* infections to virulence factors such as the biofilm, *toxR* gene, *tdh* and *trh*. Li et al. (2020) also associated biofilm formation with the *toxR* gene, a virulence factor ubiquitously present in *V. parahaemolyticus*. Similarly, Ashrafudoulla et al. (2019) stated that *V. parahaemolyticus* biofilm showed a strong genetic relationship with *tdh* gene. The study of Ashrafudoulla et al. (2019) stated that biofilm formation by food isolates could result in potential risk to consumer’s health. All isolates by Ashrafudoulla et al. (2019) showed strong biofilm formation capacity on food surfaces which was higher compared to our study. Han et al. (2016) reported that both the cultural counts and biofilm formation index values of *V. parahaemolyticus* were stronger at temperatures between 15–37°C. Han et al. (2016) finalized that exoprotease production, as well as significantly stronger biofilm formation, were produced at 25–37°C on food contact surfaces and food.

The O antigen is a crucial component of bacterial lipopolysaccharide (Samuel et al., 2004). A capsule can be produced by *V. parahaemolyticus* with the variability of the O antigen vital for bacterial classification depending on external factors. Our result showed that the O2 serovar was more compared to others detected which tallies to the findings of Xu et al. (2016); and Li et al. (2020). Also, PCR-detected serotyping of 145 isolates for O-antigen by Xu et al. (2016) revealed that serotypes with the exemption of O6, O9, and O7, were detected which was not in line with our study as O6 and O7 serotypes were detected in our study. Furthermore, our findings disagree with the study of Zhao et al. (2011) that detected the O3 serotype as the most detected serotype from shellfish and the study of Narayanan et al. (2020) that reported the prevalence O1 serotype in *V. parahaemolyticus* recovered from retail food and water samples. Location and serogroups did not correlate the phenotypic and/or genotypic profile of the *V. parahaemolyticus* in our study. Monitoring variation in serogroup could be vital in improving our understanding of food pathogens (such as *V. parahaemolyticus*) responsible for food poisoning. The O antigen of vibrios as a receptor, domiciled on the bacterial surface can be an important target for specific phages (Xu et al., 2014). This could aid the control and prevention of MDR pathogenic bacteria by phages aiming at specific O serogroup of *V. parahaemolyticus*.

A significant concern of global health has been a persistent increase in antibiotic-resistant infections. Surveillance of antibiotic-resistant bacteria and dissemination of surveillance findings are crucial in addressing this public health menace (Johnson, 2015). Findings from our study correspond with Xu et al. (2016) that reported high resistance of *V. parahaemolyticus* to ampicillin, cefazolin and cephalothin. Sudha et al. (2014) attributed the resistance of ampicillin and other first-generation antibiotics to their extensive usage in the treatment of *V. parahaemolyticus* thus the increase in resistance and resulting low efficacy. Devi et al. (2009) attributed resistance of *V. parahaemolyticus* to penicillin and other penicillin derivatives to β-lactamase that are chromosomally encoded. Similarly, Blair et al. (2014) also attributed *V. parahaemolyticus* resistance to penicillin to likely to be a result of Gram-negative bacteria complexity of their outer membrane which limits antibiotic compounds from entering the outer membrane.

Furthermore, this study levels up with the report of Letchumanan et al. (2015) where the resistance of cefotaxime and ceftazidime (third-generation cephalosporin) to *V. parahaemolyticus* isolates was reported. Similarly, Li et al. (2020) revealed a high resistance of cephalothin; a first-generation cephalosporin. Contrarily, Shaw et al. (2014) reported *V. parahaemolyticus* isolated from the United States to show less resistance to cefotaxime. Also, Hu et al. (2020) denoted that *V. parahaemolyticus* strains were highly sensitive to ceftazidime. Nevertheless, these contradictory reports on the resistance pattern of *V. parahaemolyticus* to 3rd generation cephalosporins could be as a result of a difference in test methodology employed or geographical variability. Furthermore, Beshiru et al. (2020) reported high sensitivity of *V. parahaemolyticus* recovered from ready-to-eat food to cephalothin which is contrary to this study. Shaw et al. (2014) and Tang et al. (2014) documented that the association of *V. parahaemolyticus* with a slight intermediate and resistance to amikacin should be of concern to avoid an elevated level of resistance to aminoglycosides. In this study, a significantly elevated level of amikacin resistance was observed, this occurrence should be of public health concern.

Most *V. parahaemolyticus* isolated by Lee et al. (2018) were susceptible to nalidixic acid, tetracycline, levofloxacin, gentamicin, imipenem, and sulfamethoxazole/trimethoprim which were in agreement with this study. The previous report by Shaw et al. (2014) demonstrated that all isolates of *V. parahaemolyticus* were fully susceptible to the gentamicin and thus recommended gentamicin as a drug of choice in the treatment for MDR *Vibrio* infections. The misuse and extensive usage of antibiotics are major factors contributing to the development of ARGs in bacteria and the dissemination of MDR bacterial. The MDR ratio from this study is less than those reported by Daramola et al. (2009) in which 78.9% of the isolates were multi-resistant. The MDR ratio observed from our study is lesser than those reported by Yang et al. (2017) where strains amounting to 68.38% were MDR. This is similar to the findings by Ahmed et al. (2018) where all isolates of *V. parahaemolyticus* demonstrated MDR. Generally, evaluating the trend in antimicrobial susceptibility of *V. parahaemolyticus* is of importance as the emergence of multiple drugs microbial resistant infections is a serious clinical problem.

Large proportions of strains of *V. parahaemolyticus* recovered from samples of environmental origin revealed resistance to multiple antibiotics such as ampicillin, carbenicillin, amoxicillin, cefazolin, gentamicin, cephalothin, and ceftazidime (Sudha et al., 2014; Tang et al., 2014; Yano et al., 2014). The MAR index observed in our study is not entirely different from the findings of Tan et al. (2020) in which more than 0.20 MAR indices were reported from 55.83% of the vibrios. The previous report by Gufe et al. (2019) stated that MAR index > 0.2 portrays the bacterial strain screened to have originated from sources that are of high-risk where antibiotics are used frequently. The variation in the MAR index of *V. parahaemolyticus* reported in various studies might have been influenced by the differences in resistance patterns which depend on sample sources and geographic distribution (Tunung et al., 2012; Tang et al., 2014). Inappropriate application of antibiotics for bacterial infection prophylactics and widespread of bacteria causing disease is likely an important cause of the emergence and dissemination of MDR in vibrios.

The detection of *dfr* resistance gene in this study is in line with a previous report by Beshiru et al. (2020) where the detection of *dfr*1 gene which denotes genetic tolerance of trimethoprim resistance was reported. In agreement with Hu et al. (2020), *tetB* and *blaOXA* resistance genes were not detected in this study. Common resistance genes by Jeamsripong et al. (2020) to those of our study were *tet*(A) (22.1%) and *bla*TEM (0.8%). The upsurge in the ARGs and the dissemination of antimicrobial-resistant vibrios could be due to the misuse and overuse of antibiotics (Yano et al., 2014). The significant proportion of antibiotics used currently for therapeutics and the ARGs obtained by pathogens isolated from humans all has an environmental source/origin. Current studies suggest that the functionalities of these determinants in their reservoirs may be distinct from the “weapon-shield” characteristics they carry out. Changes in ecosystems, which include the misuse/abuse of significant proportions of antimicrobials, can alter the population structure and dynamics of microorganisms, including resistance selection, with detrimental effects on human health.

## Conclusion

This study confirms that African salads are potential reservoirs for *V. parahaemolyticus* with MAR. The detection of toxigenic and ARGs of *V. parahaemolyticus* gives us comprehensive understanding regarding the occurrence of pathogenic and MDR strains of *V. parahaemolyticus* in African salads. The occurrence of resistance genetic elements of *V. parahaemolyticus* enables the possibility of horizontal dissemination of ARGs among bacteria. The findings from this study are important in defining key monitoring programs, information for risk and exposure assessment of *V. parahaemolyticus* pathogens. Furthermore, important measures which include good hygiene practices by the handlers of food materials, the use of clean preparation utensils and proper storage materials could be vital to circumvent cross-contamination.

## Data Availability Statement

The original contributions presented in the study are included in the article/Supplementary Material, further inquiries can be directed to the corresponding author/s.

## Author Contributions

EI and AB conceived, designed, and performed the experiments. EI, AB, II, AO, and KU analyzed the data. EI, AB, and II contributed reagents and materials. EI, AB, II, AO, and KU contributed to the writing of the manuscript. All authors contributed to the article and approved the submitted version.

## Conflict of Interest

The authors declare that the research was conducted in the absence of any commercial or financial relationships that could be construed as a potential conflict of interest.
