# Supplementary material for: Prevalence and Characterization of Food-Borne Vibrio parahaemolyticus From African Salad in Southern Nigeria
Source: Front Microbiol. 2021 Jun 8;12:632266. doi: 10.3389/fmicb.2021.632266 (PMC8217614; doi:10.3389/fmicb.2021.632266)
Supplement: Supplementary file 1 [file Data_Sheet_1.PDF]

Prevalence and characterization of food-borne *Vibrio parahaemolyticus* from African salad in Southern Nigeria

#Etinosa O. Igbinosa[1,*], #Abeni Beshiru[1,2], Isoken H. Igbinosa[1], Abraham G. Ogofure[1], Kate E. Uwhuba[1,2]

[1]Applied Microbial Processes & Environmental Health Research Group, Faculty of Life Sciences, University of Benin, Private Mail Bag 1154 Benin City 300283, Edo State, Nigeria

[2]Department of Microbiology, College of Natural and Applied Sciences, Western Delta University, Private Mail Bag 10 Oghara, Delta State, Nigeria

#these two **authors** contributed equally

*** Correspondence:**
Etinosa O. Igbinosa
eigbinosa@gmail.com

SUPPLEMENTARY TABLE 1. Primers used in this study

Target genes	Primer sequence	Amplicon size (bp)	Annealing condition	References
Toxin operon gene (*tox*-R)	*toxR*-F (5'-ATA CGA GTG GTT GCT GTC ATG-3')<br>*toxR*-R (5'-GTC TTC TGA CGC AAT CGT TG-3')	368	68°C for 1 min	Kim *et al*. (1999)
Thermostable direct hemolysin (*tdh*)	*tdh*-F (5'-GTA AAG GTC TCT GAC TTT TGG AC-3')<br>*tdh*-R (5'-TGG AAT AGA ACC TTC ATC TTC ACC-3')	270	58°C for 1 min	Bej *et al*. (1999)
TDH-related hemolysin (*trh*)	*trh*-F (5'-TTG GCT TCG ATA TTT TCA GTA TCT-3')<br>*trh*-R (5'-CAT AAC AAA CAT ATG CCC ATT TCC G-3')	486	58°C for 1 min	Bej *et al*. (1999)
Sulfamethoxazole (*sul3*)	*sul3*-F (5'-GAGCAAGATTTTTGGAATCG-3')<br>*sul3*-R (5'-CTAACCTAGGGCTTTGGATAT-3')	750	53°C for 40 s	Aarestrup *et al*. (2003)
Sulfamethoxazole (*sul2*)	*sul2*-F (5'-GCGCTCAAGGCAGATGGCATT-3')<br>*sul2*-R (5'-GCGTTTGATACCGGCACCCGT-3')	293	60°C for 1 min	Aarestrup *et al*. (2003)
Sulfamethoxazole (*sul1*)	*sul1*-F (5'-CTTCGATGAGAGCCGGCGGC-3')<br>*sul1*-R (5'-GCAAGGCGGAAACCCGCGCC-3')	433	60°C for 1 min	Perreten and Boerlin (2003)
Kanamycin (*aphA-3*)	*aphA*-3-F (5'-GGGACCACCTATGATGTGGAACG-3')<br>*aphA*-3-R (5'-CAGGCTTGATCCCCAGTAAGTC-3')	600	55°C for 1 min	Gibreel *et al*. (2004)
Chloramphenicol (*catA1*)	*catA1*-F (5'-CGCCTGATGAATGCTCATCCG-3')<br>*catA1*-R (5'-CCTGCCACTCATCGCAGTAC-3')	456	58°C for 1 min	Soge *et al*. (2006)
Chloramphenicol (*catA2*)	*catA2*-F (5'-ATGAATTTTACCAGAATTGATCTGAA-3')<br>*catA2*-R (5'-ATTTCAGTATGTTATCACACATCATCT-3')	639	58°C for 1 min	Soge *et al*. (2006)
Chloramphenicol (*catA3*)	*catA3*-F (5'-AAATTGGGTTCGCCGTGA-3')<br>*catA3*-R (5'-ATTTACTGTTACACAACTCTTGTAGCC-3')	1863	58°C for 1 min	Soge *et al*. (2006)
Chloramphenicol (*catB3*)	*catB3*-F (5'-TCAAAGGCAAGCTGCTTTCTGAGC-3')<br>*catB3*-R (5'-TATTAGACGAGCACAGCATGGGCA-3')	566	58°C for 1 min	Soge *et al*. (2006)
Trimethoprim (*dfr*)	*dfr17*-F (5'-GAAAATATCATTGATTTCTGCAGTG-3')<br>*dfr17*-R (5'-TTTTTCCAAATCTGGTATGTATAATTT-3')	465	60°C for 45 s	Grape *et al*. (2007)
Tetracycline (*tetA*)	*tetA*-F (5'-GTAATTCTGAGCACTGTCGC-3')<br>*tetA*-R (5'-CTGCCTGGACAACATTGCTT-3')	956	58°C for 1 min	Lucarelli *et al*. (2010)
Tetracycline (*tetB*)	*tetB*-F (5'-ACGTTACTCGATGCCAT-3')<br>*tetB*-R (5'-AGCACTTGTCTCCTGTT-3')	1169	58°C for 45 s	Lucarelli *et al*. (2010)
Tetracycline	*tetC*-F (5'-AACAATGCGCTCATCGT-3')	1138	58°C for	Lucarelli *et al*.

(*tetC*)	*tetC*-R (5’-GGAGGCAGACAAGGTAT-3’)		1 min	(2010)
Tetracycline (*tetG*)	*tetG*-F (5’-CCGGTCTTATGGGTGCTCTA-3’)<br>*tetG* -R (5’-CCAGAAGAACGAAGCCAGTC-3’)	603	58°C for 1 min	Lucarelli *et al*. (2010)
Tetracycline (*tetM*)	*tetM*-F (5’-ACAGAAAGCTTATTATATAAC-3’)<br>*tetM*-R (5’-TGGCGTGTCTATGATGTTCAC-3’)	171	55°C for 45 s	Aminov *et al*. (2001)
β-lactams (*blaTEM*)	*blaTEM* -F (5’-CATTTCCGTGTCGCCCTTAT-3’)<br>*blaTEM* -R (5’-TCCATAGTTGCCTGACTCCC-3’)	793	58°C for 1 min	Randall *et al*. (2004)
β-lactams (*blaOXA*)	*blaOXA*-F (5’-ACCAGATTCAACTTTCAA-3’)<br>*blaOXA*-R (5’-TCTTGGCTTTTATGCTTG-3’)	590	58°C for 1 min	Gallardo *et al*. (1999)
β-lactams (*blaSHV*)	*blaSHV*-F (5’-TTATCTCCCTGTTAGCCACC-3’)<br>*blaSHV*-R (5’-GATTTGCTGATTTCGCTCGG-3’)	795	58°C for 1 min	Hasman *et al*. (2005)

SUPPLEMENTARY TABLE 2. Phenotypic and genotypic virulence profile of *V. parahaemolyticus* from African salads

**Isolate code**	**Location**	**Biofilm Formation**	**Urease+ 2% NaCl**	**Hemolytic response**	***toxR*-gene**	***trh*-gene**	***tdh*-gene**	**Serogroup**
Vp003	Edo	Weak	-	-	+	-	-	O10
Vp004	Edo	Negative	-	-	+	-	-	Uncertain
Vp009	Edo	Weak	-	-	+	-	-	O12
Vp010	Delta	Strong	+	-	+	+	-	O2
Vp011	Delta	Moderate	+	+	+	+	+	O12
Vp013	Delta	Strong	+	+	+	+	+	O2
Vp014	Lagos	Weak	-	-	+	-	-	O6
Vp017	Imo	Moderate	-	-	+	-	-	O2
Vp018	Imo	Strong	+	-	+	+	-	O2
Vp019	Imo	Moderate	-	-	+	-	-	Uncertain
Vp020	Imo	Weak	-	-	+	-	-	O1
Vp023	Delta	Moderate	-	-	+	-	-	O12
Vp024	Delta	Strong	+	-	+	+	-	O2
Vp025	Delta	Strong	+	+	+	+	+	O2
Vp026	Imo	Moderate	-	-	+	-	-	O6
Vp028	Anambra	Moderate	-	-	+	-	-	O11
Vp029	Anambra	Moderate	+	-	+	+	-	O12
Vp031	Anambra	Weak	-	-	+	-	-	O6
Vp033	Delta	Moderate	-	-	+	-	-	O12
Vp034	Delta	Weak	-	-	+	-	-	O12
Vp035	Delta	Moderate	-	-	+	-	-	O2
Vp038	Delta	Weak	-	-	+	-	-	O4
Vp039	Edo	Strong	+	+	+	+	+	O1
Vp040	Edo	Moderate	-	-	+	-	-	O2
Vp043	Edo	Moderate	-	-	+	-	-	Uncertain
Vp048	Edo	Moderate	+	-	+	+	-	O2
Vp052	Ondo	Weak	-	-	+	-	-	O1
Vp053	Ondo	Negative	-	-	+	-	-	Uncertain
Vp056	Ondo	Negative	-	-	+	-	-	O7
Vp064	Lagos	Moderate	-	-	+	-	-	O1
Vp072	Enugu	Weak	-	-	+	-	-	O2
Vp073	Abia	Moderate	-	-	+	-	-	O11
Vp091	Abia	Moderate	-	-	+	-	-	O2
Vp097	Abia	Moderate	-	-	+	-	-	O10
Vp103	Abia	Strong	+	+	+	+	+	O2
Vp111	Lagos	Moderate	-	-	+	-	-	O12
Vp125	Lagos	Strong	+	-	+	+	-	O12
Vp134	Lagos	Moderate	-	-	+	-	-	O2
Vp141	Enugu	Moderate	-	-	+	-	-	O2
Vp142	Enugu	Strong	+	+	+	+	+	O12

Vp147	Lagos	Weak	-	-	+	-	-	O1
Vp149	Enugu	Moderate	-	-	+	-	-	O1
Vp150	Lagos	Strong	+	+	+	+	+	O2
Vp152	Enugu	Moderate	-	-	+	-	-	Uncertain
Vp164	Enugu	Strong	+	+	+	+	+	O12
Vp165	Lagos	Weak	-	-	+	-	-	O2
Vp171	Ondo	Negative	-	-	+	-	-	O2
Vp172	Ondo	Weak	-	-	+	-	-	O2
Vp173	Abia	Moderate	+	+	+	+	+	O2
Vp175	Abia	Moderate	-	-	+	-	-	O12
Vp177	Abia	Moderate	-	-	+	-	-	O12
Vp178	Enugu	Moderate	-	-	+	-	-	O12
Vp180	Enugu	Negative	-	-	+	-	-	O2
Vp182	Enugu	Moderate	-	-	+	-	-	O10
Vp185	Anambra	Moderate	-	-	+	-	-	Uncertain
Vp192	Anambra	Strong	+	-	+	+	-	O10
Vp194	Anambra	Moderate	-	-	+	-	-	O2
Vp195	Anambra	Strong	+	-	+	+	-	O2
Vp196	Anambra	Moderate	-	-	+	-	-	O12
Vp198	Imo	Moderate	+	-	+	+	-	O1
Vp223	Imo	Strong	+	-	+	+	-	O12
Vp224	Imo	Moderate	-	-	+	-	-	O12
Vp229	Imo	Strong	+	-	+	+	-	O4

Legend: +: positive; -: negative

SUPPLEMENTARY TABLE 3. Correlation between phenotypic and genotypic virulence profile of *V. parahaemolyticus* from African salads

**Variables**		**Location**	**Biofilm formation**	**Urease+ 2%NaCl**	**Hemolytic response**	***trh* gene**	***tdh* gene**	**Serogroup**
	Pearson Correlation	1	-.208	-.185	-.061	-.185	-.061	-.134
Location	Sig. (2-tailed)		.101	.146	.633	.146	.633	.294
	N	63	63	63	63	63	63	63
	Pearson Correlation	-.208	1	.686**	.424**	.686**	.424**	-.033
Biofilm formation	Sig. (2-tailed)	.101		.000	.001	.000	.001	.798
	N	63	63	63	63	63	63	63
	Pearson Correlation	-.185	.686**	1	.599**	1.000**	.599**	-.163
Urease+2%NaCl	Sig. (2-tailed)	.146	.000		.000	.000	.000	.202
	N	63	63	63	63	63	63	63
	Pearson Correlation	-.061	.424**	.599**	1	.599**	1.000**	-.106
Hemolytic response	Sig. (2-tailed)	.633	.001	.000		.000	.000	.409
	N	63	63	63	63	63	63	63
	Pearson Correlation	-.185	.686**	1.000**	.599**	1	.599**	-.163
trh gene	Sig. (2-tailed)	.146	.000	.000	.000		.000	.202
	N	63	63	63	63	63	63	63
tdh gene	Pearson Correlation	-.061	.424**	.599**	1.000**	.599**	1	-.106

	Sig. (2-tailed)	.633	.001	.000	.000	.000		.409
	N	63	63	63	63	63	63	63
	Pearson Correlation	-.134	-.033	-.163	-.106	-.163	-.106	1
Serogroup	Sig. (2-tailed)	.294	.798	.202	.409	.202	.409	
	N	63	63	63	63	63	63	63

**Correlation is significant at 0.01 level (2-tailed)

SUPPLEMENTARY TABLE 4. Antibiotic susceptibility breakpoints of the *V. parahaemolyticus*

**Antimicrobial class**	**Antibiotics**	**Resistance**	**Intermediate**	**Sensitive**
Penicillins	Ampicillin/sulbactam (10/10 µg)	≤11	12-14	≥15
	Ampicillin (10 µg)	≤13	14-16	≥17
Aminoglycosides	Amikacin (30 µg)	≤14	15-16	≥17
	Gentamicin (10 µg)	≤12	13-14	≥15
	Kanamycin (30 µg)	≤13	14-17	≥18
	Streptomycin (10 µg)	≤11	12-14	≥15
Carbapenems	Imipenem (10 µg)	≤19	20-22	≥23
Cephalosporins	Cefotaxime (30 µg)	≤22	23-25	≥26
	Ceftazidime (30 µg)	≤17	18-20	≥21
	Cephalothin (30 µg)	≤22	23-25	≥26
	Cefazolin (30 µg)	≤14	-	≥15
Quinolones	Nalidixic acid (30 µg)	≤13	14-18	≥19
	Levofloxacin (5 µg)	≤13	14-16	≥17
	Ciprofloxacin (5 µg)	≤15	16-20	≥21
Phenicols	Chloramphenicol (30 µg)	≤12	13-17	≥18
Folate pathway inhibitor	Trimethoprim-sulfamethoxazole (1.25/23.75 µg)	≤10	11-15	≥16
Tetracyclines	Tetracycline (30 µg)	≤11	12-14	≥15
	Oxytetracycline (30 µg)	≤11	12-14	≥15
Macrolides	Azithromycin (15 µg)	≤13	14-17	≥18

SUPPLEMENTARY TABLE 5. Diameter zone of the results of the *V. parahaemolyticus*

Isolate codes	AMP	IMI	CAZ	CTX	AMK	SAM	KZ	KF	AZM	CHL	GEN	CIP	LEV	KAN	STR	NAL	TET	OXY	STX
Vp003	14± 0.01	27± 0.07	25± 0.00	23± 0.07	18± 0.18	17± 0.01	19± 0.06	27± 0.07	21± 0.06	19± 0.09	21± 0.00	25± 0.01	23± 0.05	17± 0.14	14± 0.18	24± 0.00	19± 0.00	17± 0.14	19± 0.02
Vp192	3± 0.00	14± 0.01	11± 0.05	13± 0.05	12± 0.00	8± 0.18	12± 0.14	19± 0.00	14± 0.06	12± 0.18	10± 0.00	13± 0.14	12± 0.13	9± 0.18	12± 0.00	19± 0.13	12± 0.05	12± 0.18	11± 0.05
Vp025	2± 0.00	20± 0.18	12± 0.14	12± 0.00	11± 0.14	8± 0.01	12± 0.00	17± 0.06	14± 0.01	10± 0.00	13± 0.08	14± 0.07	10± 0.01	11± 0.07	9± 0.05	19± 0.05	12± 0.01	12± 0.00	9± 0.10
Vp011	2± 0.00	22± 0.00	12± 0.01	14± 0.18	12± 0.07	12± 0.07	10± 0.05	15± 0.05	18± 0.08	18± 0.00	14± 0.05	12± 0.06	10± 0.06	12± 0.00	8± 0.09	21± 0.07	10± 0.00	10± 0.13	12± 0.09
Vp103	4± 0.05	11± 0.00	18± 0.06	12± 0.01	10± 0.08	9± 0.05	15± 0.01	15± 0.09	15± 0.18	12± 0.01	11± 0.13	10± 0.09	11± 0.09	10± 0.08	12± 0.07	13± 0.06	13± 0.13	13± 0.09	11± 0.18
Vp150	2± 0.02	21± 0.09	14± 0.00	11± 0.01	10± 0.05	8± 0.09	12± 0.18	15± 0.18	14± 0.01	12± 0.13	14± 0.07	16± 0.14	14± 0.08	12± 0.01	12± 0.13	20± 0.09	11± 0.09	11± 0.00	9± 0.01
Vp164	3± 0.14	20± 0.00	13± 0.18	14± 0.14	9± 0.00	10± 0.01	9± 0.08	16± 0.14	19± 0.05	11± 0.01	14± 0.00	12± 0.05	10± 0.00	13± 0.01	11± 0.05	21± 0.14	13± 0.05	14± 0.02	14± 0.14
Vp177	2± 0.00	22± 0.07	12± 0.00	15± 0.09	10± 0.00	12± 0.00	12± 0.00	12± 0.01	19± 0.14	12± 0.05	13± 0.18	10± 0.13	11± 0.07	10± 0.05	13± 0.01	21± 0.00	10± 0.00	10± 0.06	15± 0.00
Vp223	5± 0.00	21± 0.05	12± 0.05	13± 0.00	11± 0.07	10± 0.06	11± 0.00	26± 0.07	14± 0.00	10± 0.01	10± 0.05	11± 0.08	12± 0.01	11± 0.09	10± 0.14	20± 0.05	12± 0.18	13± 0.10	14± 0.08
Vp010	2± 0.01	20± 0.00	14± 0.14	14± 0.07	10± 0.18	10± 0.09	10± 0.00	12± 0.00	18± 0.05	10± 0.09	11± 0.14	17± 0.07	16± 0.05	9± 0.01	11± 0.07	21± 0.08	14± 0.07	14± 0.09	15± 0.05
Vp028	4± 0.06	23± 0.00	11± 0.00	12± 0.01	12± 0.05	14± 0.01	11± 0.07	13± 0.09	19± 0.08	20± 0.00	10± 0.00	16± 0.00	15± 0.14	11± 0.00	10± 0.00	19± 0.09	11± 0.05	11± 0.00	12± 0.07
Vp033	4± 0.05	25± 0.00	10± 0.07	11± 0.08	12± 0.00	13± 0.05	10± 0.00	12± 0.08	18± 0.00	12± 0.14	13± 0.08	14± 0.06	10± 0.01	14± 0.07	8± 0.09	19± 0.18	13± 0.01	13± 0.07	10± 0.00
Vp064	3± 0.00	24± 0.02	11± 0.06	14± 0.06	11± 0.06	12± 0.07	8± 0.05	14± 0.00	18± 0.00	12± 0.01	14± 0.07	17± 0.09	14± 0.13	12± 0.06	13± 0.08	24± 0.07	10± 0.13	10± 0.05	9± 0.06
Vp229	6± 0.00	25± 0.18	11± 0.00	12± 0.01	10± 0.00	11± 0.01	9± 0.14	27± 0.06	19± 0.07	11± 0.18	13± 0.00	21± 0.00	15± 0.00	12± 0.01	12± 0.00	21± 0.13	10± 0.02	9± 0.00	9± 0.00
Vp013	7± 0.00	26± 0.00	5± 0.08	14± 0.05	10± 0.01	10± 0.08	10± 0.06	12± 0.00	18± 0.18	12± 0.09	14± 0.00	24± 0.01	16± 0.18	14± 0.05	11± 0.05	21± 0.09	14± 0.00	12± 0.14	10± 0.05
Vp018	2± 0.00	25± 0.09	18± 0.05	16± 0.14	11± 0.07	10± 0.18	16± 0.00	13± 0.18	19± 0.06	20± 0.13	13± 0.00	23± 0.05	17± 0.09	11± 0.01	10± 0.00	23± 0.10	11± 0.05	11± 0.08	11± 0.14

Vp039	3± 0.00	26± 0.13	14± 0.01	14± 0.09	10± 0.00	9± 0.09	11± 0.01	14± 0.05	20± 0.05	10± 0.01	14± 0.00	22± 0.07	17± 0.01	12± 0.14	10± 0.07	21± 0.14	15± 0.01	13± 0.09	10± 0.00
Vp052	2± 0.01	27± 0.00	12± 0.00	11± 0.01	12± 0.05	12± 0.01	17± 0.00	13± 0.00	19± 0.01	9± 0.05	15± 0.13	14± 0.01	10± 0.05	15± 0.00	11± 0.01	20± 0.05	15± 0.14	10± 0.01	15± 0.10
Vp173	2± 0.01	23± 0.01	13± 0.14	12± 0.07	19± 0.01	10± 0.06	12± 0.01	14± 0.14	12± 0.00	13± 0.01	17± 0.05	23± 0.08	19± 0.01	15± 0.18	12± 0.18	21± 0.01	10± 0.09	10± 0.07	10± 0.01
Vp195	2± 0.00	23± 0.00	12± 0.02	14± 0.01	12± 0.00	10± 0.09	12± 0.08	24± 0.07	21± 0.07	14± 0.10	9± 0.00	22± 0.00	18± 0.07	12± 0.01	13± 0.05	24± 0.02	11± 0.06	11± 0.18	15± 0.09
Vp040	2± 0.00	25± 0.00	14± 0.13	14± 0.18	10± 0.06	13± 0.07	11± 0.05	12± 0.08	19± 0.14	15± 0.00	15± 0.00	24± 0.00	19± 0.00	14± 0.07	10± 0.00	21± 0.01	10± 0.07	10± 0.00	12± 0.18
Vp125	15± 0.01	23± 0.09	12± 0.01	16± 0.00	10± 0.08	9± 0.01	12± 0.18	14± 0.00	20± 0.01	14± 0.09	17± 0.18	24± 0.01	17± 0.00	16± 0.01	13± 0.07	20± 0.18	10± 0.01	9± 0.13	10± 0.01
Vp019	4± 0.08	24± 0.01	19± 0.18	9± 0.01	10± 0.05	12± 0.18	12± 0.01	13± 0.14	18± 0.18	16± 0.01	16± 0.07	14± 0.13	17± 0.14	17± 0.05	10± 0.01	23± 0.07	15± 0.05	14± 0.01	11± 0.00
Vp020	16± 0.07	24± 0.00	20± 0.05	25± 0.01	18± 0.07	14± 0.09	16± 0.07	24± 0.00	20± 0.00	13± 0.05	19± 0.14	25± 0.09	20± 0.05	15± 0.13	12± 0.10	21± 0.01	16± 0.00	13± 0.10	14± 0.05
Vp024	3± 0.00	26± 0.07	18± 0.01	12± 0.05	12± 0.00	9± 0.01	11± 0.08	13± 0.09	19± 0.01	15± 0.00	16± 0.01	23± 0.06	21± 0.01	14± 0.09	10± 0.01	20± 0.14	17± 0.10	12± 0.09	13± 0.01
Vp029	3± 0.00	25± 0.01	10± 0.08	10± 0.01	11± 0.14	14± 0.06	17± 0.01	15± 0.00	21± 0.05	16± 0.01	21± 0.13	22± 0.00	20± 0.13	15± 0.01	9± 0.09	21± 0.05	17± 0.01	9± 0.05	11± 0.14
Vp048	3± 0.05	24± 0.10	11± 0.07	10± 0.02	11± 0.18	12± 0.05	17± 0.06	15± 0.00	20± 0.01	16± 0.06	21± 0.00	23± 0.01	22± 0.07	15± 0.00	8± 0.02	22± 0.08	17± 0.01	10± 0.07	10± 0.08
Vp031	16± 0.00	24± 0.09	19± 0.01	24± 0.09	18± 0.05	13± 0.01	16± 0.05	23± 0.07	20± 0.01	17± 0.14	16± 0.14	22± 0.01	19± 0.00	14± 0.07	13± 0.14	20± 0.01	18± 0.18	12± 0.00	12± 0.01
Vp034	4± 0.06	25± 0.01	12± 0.14	15± 0.07	12± 0.01	12± 0.07	12± 0.10	15± 0.01	19± 0.07	14± 0.00	18± 0.01	25± 0.00	19± 0.06	18± 0.05	13± 0.01	23± 0.13	18± 0.00	13± 0.01	14± 0.06
Vp091	5± 0.14	25± 0.00	13± 0.05	17± 0.01	11± 0.13	17± 0.09	10± 0.00	13± 0.06	11± 0.06	15± 0.08	18± 0.07	23± 0.07	21± 0.08	19± 0.10	12± 0.00	24± 0.07	17± 0.07	13± 0.14	16± 0.10
Vp141	5± 0.01	24± 0.08	20± 0.01	15± 0.14	12± 0.00	15± 0.01	17± 0.00	12± 0.05	18± 0.01	16± 0.18	20± 0.01	25± 0.00	20± 0.01	19± 0.00	12± 0.06	21± 0.01	10± 0.05	10± 0.01	10± 0.14
Vp178	4± 0.00	25± 0.00	10± 0.00	14± 0.00	19± 0.07	9± 0.00	11± 0.00	14± 0.00	12± 0.10	14± 0.05	19± 0.05	22± 0.05	20± 0.00	15± 0.09	13± 0.07	20± 0.01	21± 0.13	14± 0.06	17± 0.00
Vp182	4± 0.18	24± 0.01	9± 0.10	14± 0.00	19± 0.02	8± 0.05	10± 0.05	14± 0.09	12± 0.01	14± 0.13	19± 0.18	23± 0.01	20± 0.18	15± 0.08	13± 0.01	21± 0.05	20± 0.14	14± 0.01	17± 0.09
Vp035	5± 0.00	23± 0.00	19± 0.01	16± 0.14	10± 0.06	16± 0.01	18± 0.07	15± 0.14	12± 0.14	14± 0.01	20± 0.10	22± 0.09	23± 0.00	18± 0.02	10± 0.14	23± 0.14	19± 0.00	12± 0.08	17± 0.05

Vp073	3± 0.00	25± 0.05	10± 0.07	14± 0.01	11± 0.10	17± 0.08	10± 0.01	15± 0.18	19± 0.07	15± 0.09	21± 0.01	24± 0.06	22± 0.13	16± 0.07	14± 0.01	21± 0.00	16± 0.08	14± 0.01	16± 0.00
Vp097	3± 0.05	26± 0.09	9± 0.01	14± 0.06	10± 0.05	17± 0.18	9± 0.06	15± 0.01	19± 0.18	15± 0.08	22± 0.00	26± 0.01	23± 0.09	16± 0.05	14± 0.05	22± 0.01	16± 0.09	14± 0.00	16± 0.00
Vp134	3± 0.07	26± 0.01	9± 0.05	12± 0.09	10± 0.08	17± 0.01	18± 0.05	15± 0.08	11± 0.00	16± 0.01	19± 0.00	22± 0.07	20± 0.05	17± 0.00	12± 0.01	20± 0.09	16± 0.18	17± 0.05	19± 0.18
Vp142	15± 0.01	24± 0.00	10± 0.18	16± 0.00	18± 0.01	10± 0.13	10± 0.14	16± 0.07	20± 0.01	15± 0.06	20± 0.13	25± 0.08	21± 0.01	17± 0.13	12± 0.09	21± 0.14	19± 0.01	10± 0.07	19± 0.07
Vp147	2± 0.00	24± 0.00	12± 0.01	17± 0.08	18± 0.00	17± 0.07	11± 0.01	14± 0.05	12± 0.05	14± 0.00	18± 0.01	23± 0.05	20± 0.07	16± 0.01	13± 0.18	20± 0.01	21± 0.07	15± 0.09	16± 0.08
Vp017	2± 0.00	25± 0.08	19± 0.00	14± 0.01	19± 0.07	18± 0.09	10± 0.08	15± 0.09	11± 0.00	13± 0.13	18± 0.07	22± 0.00	20± 0.10	15± 0.14	12± 0.00	19± 0.07	19± 0.00	17± 0.00	18± 0.05
Vp023	2± 0.00	24± 0.00	20± 0.01	15± 0.10	10± 0.00	17± 0.02	19± 0.18	12± 0.00	20± 0.01	15± 0.14	19± 0.08	24± 0.13	23± 0.01	17± 0.06	9± 0.14	21± 0.05	16± 0.01	16± 0.18	18± 0.14
Vp198	4± 0.13	25± 0.07	19± 0.00	15± 0.05	19± 0.14	17± 0.05	18± 0.05	24± 0.00	12± 0.06	20± 0.09	21± 0.05	22± 0.09	21± 0.02	17± 0.05	12± 0.07	21± 0.00	17± 0.01	17± 0.14	16± 0.00
Vp004	2± 0.00	23± 0.05	25± 0.07	17± 0.01	15± 0.05	18± 0.10	18± 0.01	14± 0.14	19± 0.08	19± 0.05	25± 0.01	24± 0.07	23± 0.14	16± 0.00	14± 0.00	19± 0.01	16± 0.10	14± 0.09	17± 0.10
Vp009	2± 0.00	24± 0.09	24± 0.01	17± 0.07	15± 0.06	18± 0.07	18± 0.07	14± 0.07	19± 0.14	19± 0.00	23± 0.00	23± 0.06	25± 0.05	16± 0.18	14± 0.08	19± 0.01	16± 0.05	14± 0.00	17± 0.09
Vp014	2± 0.00	24± 0.01	25± 0.00	25± 0.09	15± 0.01	18± 0.00	17± 0.06	23± 0.01	20± 0.18	20± 0.08	20± 0.18	25± 0.01	25± 0.00	23± 0.07	14± 0.05	23± 0.18	19± 0.01	18± 0.13	18± 0.06
Vp026	3± 0.05	23± 0.00	24± 0.18	23± 0.01	16± 0.18	17± 0.14	16± 0.01	24± 0.06	18± 0.00	19± 0.06	17± 0.01	23± 0.18	19± 0.07	21± 0.00	12± 0.14	21± 0.01	16± 0.09	17± 0.05	18± 0.00
Vp038	2± 0.00	25± 0.01	25± 0.00	24± 0.18	15± 0.01	17± 0.18	16± 0.05	25± 0.09	19± 0.01	21± 0.14	18± 0.13	21± 0.08	19± 0.01	20± 0.08	13± 0.00	20± 0.01	19± 0.00	17± 0.07	19± 0.07
Vp043	2± 0.01	24± 0.00	26± 0.01	23± 0.00	15± 0.00	18± 0.00	17± 0.02	24± 0.10	18± 0.14	21± 0.00	18± 0.00	22± 0.01	21± 0.08	21± 0.09	12± 0.07	23± 0.07	19± 0.01	18± 0.00	17± 0.05
Vp053	3± 0.10	23± 0.01	25± 0.14	25± 0.01	16± 0.08	16± 0.06	16± 0.13	23± 0.01	20± 0.07	20± 0.13	20± 0.14	23± 0.05	20± 0.06	22± 0.01	14± 0.06	24± 0.05	17± 0.14	17± 0.01	18± 0.00
Vp056	2± 0.00	25± 0.00	24± 0.01	25± 0.05	15± 0.05	17± 0.05	17± 0.01	23± 0.05	20± 0.05	19± 0.05	19± 0.07	22± 0.02	20± 0.00	20± 0.05	14± 0.09	23± 0.14	19± 0.01	16± 0.05	17± 0.00
Vp072	2± 0.00	24± 0.01	25± 0.00	26± 0.14	16± 0.01	17± 0.08	16± 0.14	27± 0.00	19± 0.01	21± 0.09	21± 0.05	23± 0.07	21± 0.01	21± 0.06	16± 0.00	24± 0.06	18± 0.01	18± 0.10	19± 0.00
Vp111	2± 0.00	23± 0.05	24± 0.07	26± 0.01	15± 0.07	16± 0.14	17± 0.00	28± 0.01	19± 0.14	22± 0.00	20± 0.01	23± 0.09	22± 0.07	20± 0.00	15± 0.00	21± 0.01	18± 0.05	15± 0.14	18± 0.08

Vp149	4± 0.07	24± 0.00	25± 0.05	27± 0.05	16± 0.01	18± 0.00	17± 0.07	27± 0.02	20± 0.07	21± 0.08	19± 0.14	24± 0.01	21± 0.00	21± 0.14	16± 0.18	19± 0.13	19± 0.00	19± 0.01	17± 0.18
Vp165	4± 0.01	24± 0.07	25± 0.00	26± 0.01	16± 0.14	17± 0.09	17± 0.18	27± 0.08	20± 0.00	20± 0.18	20± 0.02	21± 0.13	22± 0.05	22± 0.07	15± 0.01	21± 0.00	16± 0.13	18± 0.18	19± 0.09
Vp172	2± 0.01	25± 0.09	24± 0.08	26± 0.14	15± 0.01	17± 0.14	16± 0.00	28± 0.09	19± 0.01	20± 0.14	18± 0.00	24± 0.00	20± 0.14	20± 0.01	17± 0.05	23± 0.14	17± 0.09	17± 0.00	18± 0.14
Vp175	3± 0.07	24± 0.01	23± 0.06	26± 0.07	15± 0.18	18± 0.07	16± 0.06	27± 0.05	21± 0.01	21± 0.00	20± 0.06	23± 0.10	18± 0.00	19± 0.05	17± 0.09	20± 0.01	19± 0.07	17± 0.07	18± 0.06
Vp180	5± 0.07	25± 0.08	23± 0.00	27± 0.09	16± 0.05	17± 0.00	17± 0.05	28± 0.02	20± 0.05	20± 0.13	20± 0.00	22± 0.05	19± 0.13	19± 0.14	15± 0.14	21± 0.07	20± 0.18	16± 0.05	19± 0.00
Vp185	2± 0.00	23± 0.05	24± 0.07	27± 0.01	15± 0.00	18± 0.18	16± 0.00	28± 0.13	19± 0.01	23± 0.05	21± 0.13	25± 0.00	21± 0.00	20± 0.09	17± 0.07	21± 0.01	19± 0.00	17± 0.09	17± 0.05
Vp194	2± 0.00	25± 0.14	23± 0.14	26± 0.01	16± 0.07	16± 0.05	16± 0.18	28± 0.00	20± 0.07	22± 0.02	21± 0.07	23± 0.07	21± 0.07	20± 0.01	16± 0.06	20± 0.06	20± 0.13	18± 0.00	17± 0.01
Vp196	2± 0.00	24± 0.01	25± 0.18	26± 0.05	16± 0.08	17± 0.01	16± 0.07	27± 0.14	19± 0.18	21± 0.08	21± 0.01	23± 0.09	21± 0.08	21± 0.18	14± 0.01	23± 0.00	16± 0.05	14± 0.01	18± 0.07
Vp224	3± 0.01	24± 0.00	24± 0.05	27± 0.07	15± 0.01	17± 0.00	17± 0.08	27± 0.09	20± 0.01	21± 0.18	19± 0.18	22± 0.00	20± 0.01	19± 0.07	12± 0.09	21± 0.01	19± 0.01	12± 0.10	17± 0.13
Vp152	2± 0.00	23± 0.01	25± 0.08	26± 0.18	16± 0.18	17± 0.14	17± 0.01	28± 0.07	20± 0.13	23± 0.00	18± 0.05	24± 0.01	19± 0.06	20± 0.05	13± 0.08	22± 0.18	18± 0.14	12± 0.01	17± 0.00
Vp171	16± 0.05	26± 0.06	20± 0.00	27± 0.01	15± 0.05	18± 0.14	15± 0.06	24± 0.18	20± 0.02	22± 0.05	24± 0.00	24± 0.08	24± 0.14	23± 0.01	17± 0.05	24± 0.09	19± 0.00	17± 0.08	18± 0.01

Legend: Values are in duplicate mean ± standard deviations. **AMP:** Ampicillin (10µg), **IMI:** Imipenem (10µg), **CAZ:** Ceftazidime (30µg), **CTX:** Cefotaxime (30µg), **AMK:** Amikacin (30µg), **SAM:** Ampicillin/sulbactam (30µg), **KZ:** Cefazolin (30µg), **KF:** Cephalothin (30µg), **AZM:** Azithromycin (15µg), **CHL:** Chloramphenicol (30µg), **GEN:** Gentamicin (10µg), **CIP:** Ciprofloxacin (5µg), **LEV:** Levofloxacin (5µg), **KAN:** Kanamycin (30µg), **STR:** Streptomycin (10µg), **NAL:** Nalidixic acid (30µg), **TET:** Tetracycline (30µg), **OXY:** Oxytetracycline (30µg), and **STX:** Trimethoprim-sulfamethoxazole (1.25/23.75µg).

References

Aarestrup, F.M., Lertworapreecha, M., Evans, M.C., Bangtrakulnonth, A., Chalermchaikit, T., and Hendriksen, R.S. (2003). Antimicrobial susceptibility and occurrence of resistance genes among *Salmonella enterica* serovar Weltevreden from different countries. J. Antimicrob. Chemother. 54, 715-718.

Aminov, R.I., Garrigues-Jeanjean, N., and Mackie, R.I. (2001). Molecular ecology of tetracycline resistance: Development and validation of primers for detection of tetracycline resistance genes encoding ribosomal protection proteins. Appl. Environ. Microbiol. 67(1), 22-32.

Bej, A.K., Patterson, D.P., Brasher, C.W., Vickery, M.C., Jones, D.D., and Kaysner, C.A. (1999). Detection of total and hemolysin-producing *Vibrio parahaemolyticus* in shell fish using multiplex PCR amplification of *tl*, *tdh* and *trh*. J. Microbiol. Meth. 36, 215-225.

Gallardo, F., Ruiz, J., Marco, F., Towner, K.J., and Vila, J. (1999). Increase in incidence of resistance to ampicillin, chloramphenicol and trimethoprim in clinical isolates of *Salmonella* serotype Typhimurium with investigation of molecular epidemiology and mechanisms of resistance. J. Med. Microbiol. 48(4), 367-374.

Gibreel, A., Skold, O., and Taylor, D.E. (2004). Characterization of plasmid-mediated *aphA*-3 kanamycin resistance in *Campylobacter jejuni*. Microb. Drug Resist. 10, 98-105.

Grape, M., Motakefi, A., Pavuluri, S., and Kahlmeter, G. (2007). Standard and real-time multiplex PCR methods for detection of trimethoprim resistance dfr genes in large collections of bacteria. Clin. Microbiol. Infect. 13, 1112-1118.

Hasman, H., Mevius, D., Veldman, K., Olesen, I., and Aarestrup, F.M. (2005). Beta-lactamases among extended-spectrum beta-lactamase (ESBL)-resistant *Salmonella* from poultry, poultry products and human patients in The Netherlands. J. Antimicrob. Chemother. 56(1), 115-121.

Kim, Y.B., Okuda, J., Matsumoto, C., Takahashi, N., Hashimoto, S., and Nishibuchi, M. (1999). Identification of *Vibrio parahaemolyticus* strains at the species level by PCR targeted to the *toxR* gene. J. Clin. Microbiol. 37, 1173-1177.

Lucarelli, C., Dionisi, A.M., Torpdahl, M., Villa, L., and Graziani, C. (2010). Evidence for a second genomic island conferring multidrug resistance in a clonal group of strains of *Salmonella enterica* serovar Typhimurium and its monophasic variant circulating in Italy, Denmark, and the United Kingdom. J. Clin. Microbiol. 48, 2103-2109.

Perreten, V., and Boerlin, P. (2003). A new sulfonamide resistance gene (*sul3*) in *Escherichia coli* is widespread in the pig population of Switzerland. Antimicrob. Agents Chemother. 47, 1169-1172.

Randall, L.P., Cooles, S.W., Osborn, M.K., Piddock, L.J.V., and Woodward, M.J. (2004). Antibiotic resistance genes, integrons and multiple antibiotic resistance in thirty-five serotypes of *Salmonella enterica* isolated from humans and animals in the UK. J. Antimicrob. Chemother. 53(2), 208-216

Soge, O. O., Adenyi, B. A. and Roberts, M. C. (2006). New antibiotic resistance genes associated with CTX-M plasmids from uropathogenic Nigerian *Klebsiella pneumoniae*. J. Antimicrob. Chemother. 58, 1048-1053.
